# Characterizing Food Policy Councils’ Network Partnerships and COVID-19 Responses

**DOI:** 10.3390/nu16070915

**Published:** 2024-03-22

**Authors:** Yeeli Mui, Atif Adam, Raychel Santo, Karen Bassarab, Julia A. Wolfson, Anne Palmer

**Affiliations:** 1 Department of International Health, Johns Hopkins Bloomberg School of Public Health, Baltimore, MD 21205, USA; aadam1@jhu.edu (A.A.); jwolfso7@jhu.edu (J.A.W.); 2 Johns Hopkins Center for a Livable Future, Department of Environmental Health and Engineering, Johns Hopkins Bloomberg School of Public Health, Baltimore, MD 21205, USA; rsanto1@jhu.edu (R.S.); kbanks10@jhu.edu (K.B.); apalmer6@jhu.edu (A.P.); 3 Department of Health Policy and Management, Johns Hopkins Bloomberg School of Public Health, Baltimore, MD 21205, USA

**Keywords:** food policy councils, food security, social network analysis, racial equity, social equity

## Abstract

The COVID-19 pandemic pushed millions of Americans into food insecurity. Food policy councils (FPCs) across the country played a vital role in organizing coordinated food responses across multiple sectors. We used a social network analysis (SNA) approach to investigate: (1) the network of partnering organizations and agencies within FPCs; (2) how the characteristics of FPCs’ network partnerships (i.e., degree, coreness, and density) related to programmatic, policy, and advocacy actions in response to the pandemic; and (3) how FPCs’ use of a racial or social equity framework shifted their network partnerships and responses. Local government agencies and food supply chain actors were core in FPCs’ network partnerships, while public utilities, correctional facilities, social justice groups, and others were non-core partners. Network density was more likely to be associated with any action by FPCs, and it was especially pronounced for advocacy actions taken by FPCs; trends were similar among FPCs that reported using a racial or social equity framework. The findings begin to uncover core actors in FPCs’ partnerships and opportunities to establish new partnerships, particularly with social justice groups. The results also suggest that network density (interconnectedness) may be more important than other network characteristics when responding to food-related needs.

## 1. Introduction

Millions of Americans were pushed into food insecurity because of the COVID-19 pandemic, which disrupted food supply chains and exposed food system vulnerabilities [1,2,3,4]. Defined as limited or uncertain access to sufficient, nutritious food for an active, healthy life, the salience of food insecurity has been underscored by its links to multiple short- and long-term health consequences, including decreased diet quality, increased frequency of snacking, poorer overall mental health, increased risk of chronic disease, and loneliness among older adults [5,6]. Among marginalized racial and ethnic groups, the burden of food insecurity is even greater. For example, in the first year of the COVID-19 pandemic in the United States, the Black–White disparity and Latinx–White disparity in food insecurity increased by 3.5 and 2.4 percentage points, respectively [7].

Addressing the multifaced challenges of food insecurity requires coordinated efforts of many diverse stakeholders and cross-sectors, from agricultural production to storage and distribution, and retail and marketing [8,9,10]. In response to heightened food needs, nonprofit organizations, grassroots coalitions, private industry, and policymakers across the country have implemented a wide range of food programs and policies. In particular, food policy councils (FPCs) have played a critical role in organizing coordinated efforts across multiple sectors to address food systems’ challenges during the COVID-19 pandemic [11]. FPCs are an “organized group of stakeholders that may be sanctioned by a government body, or exist independent of government, which work to address food systems issues and needs at the local (city/municipality or county), state/provincial, regional, or tribal levels” [12]. Representing different food system and non-food system sectors, FPC membership comprises stakeholders such as representatives from food banks or other charitable feeding programs, public health practitioners, local and state government representatives, interested citizens, regional farmers, and large-scale purchasers. Key to FPCs is their use of a food system approach whereby FPCs focus on a variety of interconnected food issues and are not limited to one specific area of concern, such as nutrition or agriculture, isolated from its broader context [13]. As a result, FPCs play an essential role in convening diverse actors to promote healthy local and regional food systems—and increasingly, they are taking deliberate action to foster racial and social equity [11]. 

A dramatic growth in the number of FPCs to a roughly estimated 450 councils and similar food policy groups in industrialized countries has been accompanied by an increasing academic interest in FPCs over the last two decades [14,15]. Prior studies have evaluated FPC activities, their membership and partners, barriers to FPC effectiveness, and facilitators that support FPC effectiveness [16]. However, gaps remain that could further the influence of FPCs. A recent scoping review determined that research on FPCs thus far has been limited by methodological gaps, often employing a case study approach with primarily qualitative methods such as interviews, focus groups, and document reviews [16]. Further, key topic areas of needed research include documenting the impact of FPC activities and more direct connections to dismantling racism. 

### A Network Approach to Strengthen Food System Responses

In the wake of the COVID-19 pandemic, strengthening food systems will, in part, require a clearer understanding of how FPCs can leverage their network partnerships to advance more resilient and sustainable local and regional food systems. The present study addresses some of the aforementioned research gaps by using a social network analysis (SNA) approach to explore the network structure (i.e., partnership patterns) of FPCs and evaluate its association with responses to the pandemic. 

In the broadest sense, SNA is an approach that focuses on the relationships among a set of individuals, groups, and/or organizations [17]. Tracing back to several disciplines, including mathematics, anthropology, and sociology, SNA has become more widely used in public health settings over the last three decades to investigate disease transmission networks [18,19], collaborative networks among community organizations [20], network influences on health behavior (e.g., physical activity) [21], social capital and social support [22], coalition building around policy [23,24], and pandemic preparedness [25]. Rather than studying the characteristics of individuals (e.g., income, gender) in relation to social processes, SNA focuses on the network structure as a whole, patterns in relationships, and their benefits [26]. 

SNA often involves mapping a social network as a group of actors (i.e., an individual person or a group of people such as an organization) whose relationships are represented by the lines between them [27]. In addition to graphical depictions of networks, network properties are useful to analyze to gain insight into the role of individual actors and subgroups in a network as well as its overall structure. Degree is a standard network measure that can be used to describe connectivity in a network by determining the number of connections per actor, such as connections to friends that might influence individuals’ health and prosocial behaviors [28] or connections to multi-sector partners that could impact the success of a public health program intervention among participating organizations [20]. 

Another widely used measure of interconnectedness within a network is density, which represents the proportion of relationships in a network relative to the number of all possible relationships. Because network density can influence how public health work is implemented (e.g., sharing information, resources, working together), it is considered potentially one of the most informative network measures when examining public systems [29]. Indeed, all actors within networks are not necessarily equal in terms of influence, and SNA can help reveal strengths or gaps in network structures, further informing decision making around partnership building to improve network function. In this sense, another network measure, centrality, can determine whether certain actors form a network’s core (i.e., coreness), while others remain at the periphery with less prominence in a network [26]. 

In food-system-related scholarship, researchers have largely employed SNA to examine producer–consumer or producer–producer interactions in local agri-food systems [30,31,32,33]. Far fewer studies have used SNA to document the relationships among food policy actors. One study assessed the networks of policy entrepreneurs addressing food insecurity in Canada [34], and another the networks between cities based on shared urban food policy declarations [35]. Though SNA is a compelling technique, it has been underutilized to better understand the structure of FPCs’ network partnerships and how different constellations may relate to changes in food policy and action. The current study seeks to address this gap by exploring the following research aims:

1. Characterize FPCs’ network partnerships;
2. Evaluate how the characteristics of FPCs’ network partnerships relate to programmatic, policy, and advocacy actions in response to the pandemic;
3. Evaluate how FPCs’ use of a racial/social equity framework shifts their network partnerships and related programmatic, policy, and advocacy actions.

## 2. Materials and Methods

### 2.1. Context 

This study uses data from the Food Policy Networks project housed at the Johns Hopkins Center for Livable Future (CLF). Since 2013, the Project has conducted a survey of FPCs across the United States to document their work. The 2020 survey included questions asked in previous years as well as additional questions to capture FPCs’ responses and adaptations to emerging food systems’ challenges during the COVID-19 pandemic. During these unprecedented challenges of the pandemic, FPCs and similar groups have been convening strategic partners, matchmaking to connect supplies with needs, communicating about available resources, and advocating for policy changes. In some instances, the role of FPCs has expanded to include new functions and areas of focus, such as advocating for state policies that provide free childcare to food retail workers. 

### 2.2. Sample

**Figure 1:**
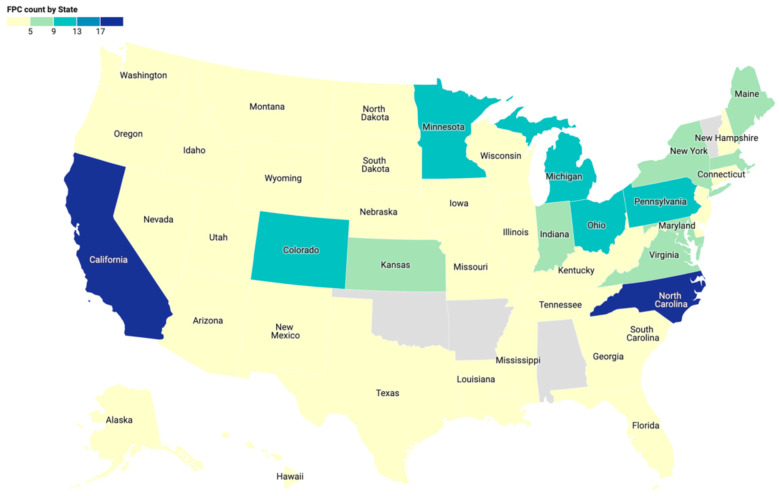
Map of surveyed FPCs across the United States.

The empirical analysis is based on primary data collection using CLF’s survey of FPCs, which was distributed to 372 FPCs and state conveners of FPCs between June and September 2020. While distributing the survey, it became evident that nearly 46 FPCs had become inactive over the past two years or did not qualify as an FPC. The survey response rate was 57.5%, and this study was based on complete responses from 195 FPCs out of the 214 responses received. The FPCs were geographically distributed around the United States and included both rural and urban areas (Figure 1). 

### 2.3. Data Collection

The survey of FPCs gathered information in two parts: (1) the structure of the FPC (e.g., geographic focus, sectors represented by membership, relationship to government); and (2) the work of the FPC in response to COVID-19, including programmatic, policy, and advocacy actions; related partnerships; and use of a racial or social equity framework in decision making (Supplementary Materials). The survey was distributed by reaching out to each FPC’s key contacts through email with a link to the web-based questionnaire system (Qualtrics, Provo, UT, USA). FPCs that did not respond to the original email received two follow-up reminder emails and a phone call from a member of the CLF team. 

#### 2.3.1. FPCs’ Network Partnerships

To identify FPC partnerships, the respondents were asked whether they had worked with various organizations or agencies on food systems concerns related to COVID-19 and were provided with a list of response options; the respondents were then asked whether each respective partner was a new or existing relationship. 

#### 2.3.2. FPCs’ Responses to the Pandemic

To determine FPCs’ responses to the pandemic, respondents were presented with separate charts of response options related to programmatic actions, policy changes, and advocacy actions. From the charts, respondents were asked to identify programmatic actions that they had led or supported, policy changes that they had led or supported in various areas of food systems work, and advocacy actions taken. Examples of programmatic actions led or supported by FPCs included creating a dashboard to track food-system-related trends in response to COVID-19, using social media (e.g., Twitter and Facebook) to share real-time information about the status of food systems resources during COVID-19, and facilitating connections across food systems sectors to match resources with needs to respond to COVID-19. Policy changes in various areas of food system work for which FPCs helped to develop or support included those impacting food chain workers and other essential workers (e.g., food service workers), federal food and nutrition assistance programs, and funding for critical food systems’ needs. Advocacy actions taken included providing policy recommendations to policy makers, submitting written testimony, and making calls to policy makers. Respondents also had the option of describing their actions if they were not captured in the charts provided. This study was deemed exempt from human subjects’ research oversight by the Bloomberg School of Public Health Institutional Review Board. 

### 2.4. Data Analysis 

#### 2.4.1. Sociograms of FPCs’ Network Partnerships

We used an SNA approach to understand which partners were central or influential to FPCs’ network and food systems responses. Using UCINET [36], we created sociograms to visually map FPCs’ relationships with partnering organizations and agencies. 

#### 2.4.2. Statistical Analysis

We used statistical models to assess the relationship between the characteristics of FPCs’ network partnerships and their food systems responses (i.e., programmatic actions led or supported, policy changes led or supported, and advocacy actions taken). The characteristics of FPCs’ network partnerships included the following independent variables:

1. Degree (number of relationships between FPCs and partner organizations or agencies). Network size is associated with measures of network density and coreness [37,38].
2. Coreness (number of relationships between FPCs and central “core” organizations or agencies). A network’s core comprises embedded, highly connected actors important to the network structure, while a network’s “periphery” comprises less connected actors.
3. Density (number of relationships with organizations or agencies relative to all possible relationships with organizations and agencies). Network density represents interconnectedness in the network, i.e., how connected the network is compared to how connected it could be (0 to 100%).

Dependent variables in our analysis were responses to survey questions about FPCs’ actions in response to the pandemic. We quantified the number of programmatic actions led/supported by FPCs (maximum of 13), the number of policy changes led/supported by FPCs (maximum of 15), and the number of advocacy actions taken by FPCs in response to the pandemic (maximum of 13). 

Because the readiness of FPCs to respond to the pandemic may have been influenced by how established the relationships were between FPCs and their partners, we categorized FPCs’ network partnerships in our statistical analysis into: (1) FPCs’ *new* networks, which comprised new relationships that were formed with organizations and agencies during the pandemic; and (2) FPCs’ *established* networks, which comprised established relationships with organizations and agencies, i.e., FPCs had previously worked and currently worked with the respective partner at the time of the survey.

We used negative binomial regression models to evaluate the association between the characteristics of FPCs’ network partnerships and food system responses. All models were adjusted for annual budget, type of FPC organization (e.g., nonprofit, embedded in government), regional location of FPC, and year of formation. Finally, separate regression models were conducted, focusing on a subset of FPCs that reported using a racial or social equity framework to guide their food systems responses; this subset of FPCs did not include those who reported developing a framework. All analyses were performed using STATA version 15.1 (StataCorp. 2017. Stata Statistical Software: Release 15. College Station, TX, USA: StataCorp LLC).

## 3. Results

In our sample of FPCs, nearly 33% were located in the Midwest, while about 25% were located in the South and West each (Table 1). The most common FPC organization types were housed in nonprofit organizations (33%) and embedded within government agencies (25%). Regarding geographic focus, 37% of FPCs worked at the county level, with 31% working at the city/municipality or the city/municipality and county levels, and the remaining 31% working at other levels. Eighty-nine FPCs (45%) operated with annual budgets of under USD 25,000, while 11% reported budgets of over USD 100,000. The FPCs reported an average of 1.89 (SD = 2.93) relationships in new networks and 9.85 (SD = 5.56) relationships in established networks.

### 3.1. Overall Sample of FPCs 

#### 3.1.1. Density and Core Relationships in FPCs’ New and Established Networks

**Figure 2:**
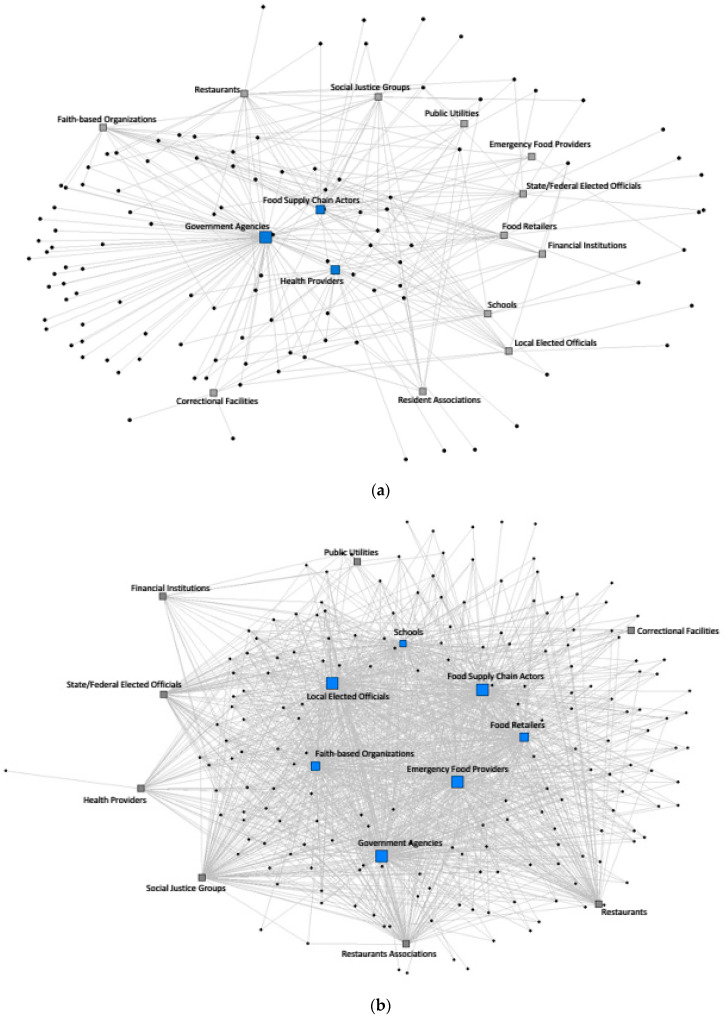
(**a**) Sociogram of core (blue) and peripheral (grey) partners within FPCs’ new networks. (**b**) Sociogram of core and peripheral partners within FPCs’ established networks.

Figure 2a,b show sociograms of FPCs’ new and established networks with partners (i.e., organizations or agencies). We observed that network density (interconnectedness) in FPCs’ new networks was 9.1% (Figure 2a) versus 42.1% in FPCs’ established networks (Figure 2b). Core partners (blue squares) in FPCs’ new networks included government agencies, food supply chain actors, and healthcare providers. In contrast, core partners in FPCs’ established networks were more expansive, including faith-based organizations, food retail stores, local elected officials, government agencies, emergency food providers, food supply chain actors, and schools. Non-core partners (i.e., peripheral actors; grey squares) in both networks included public utilities, correctional facilities, social justice groups, banks/financial institutions, and resident associations. 

#### 3.1.2. Characteristics of FPCs’ Network Partnerships Associated with Programmatic, Policy, and Advocacy Actions

Compared to their new networks, the characteristics within FPCs’ established networks were associated with a greater likelihood of being responsive to food systems’ needs (Table 2). Specifically, within FPCs’ established networks, increases in all network characteristics—degree, coreness, and density—were significantly associated with a greater likelihood of FPCs leading or supporting programmatic actions, leading or supporting policy changes, and taking on advocacy actions. The greatest effect size was observed in relation to network density. For example, we found that a one-unit increase in network density within FPCs’ established networks was associated with a 2.37 times greater likelihood of leading or supporting programmatic actions (95% CI 1.58, 3.57; *p* < 0.01). Within FPCs’ new networks, for every unit increase in degree, coreness, and density, we found an increased likelihood of leading or supporting programmatic actions only; we did not observe significant associations in relation to leading or supporting policy changes or advocacy actions taken. Again, network density had the largest association, whereby every unit increase was associated with a 1.73 times greater likelihood of leading or supporting programmatic actions within FPCs’ new networks (95% CI 1.14, 2.61; *p* < 0.05).

### 3.2. Subset of FPCs Using a Racial or Social Equity Framework 

#### 3.2.1. Density and Core Relationships of FPCs’ Using a Racial or Social Equity Framework

**Figure 3:**
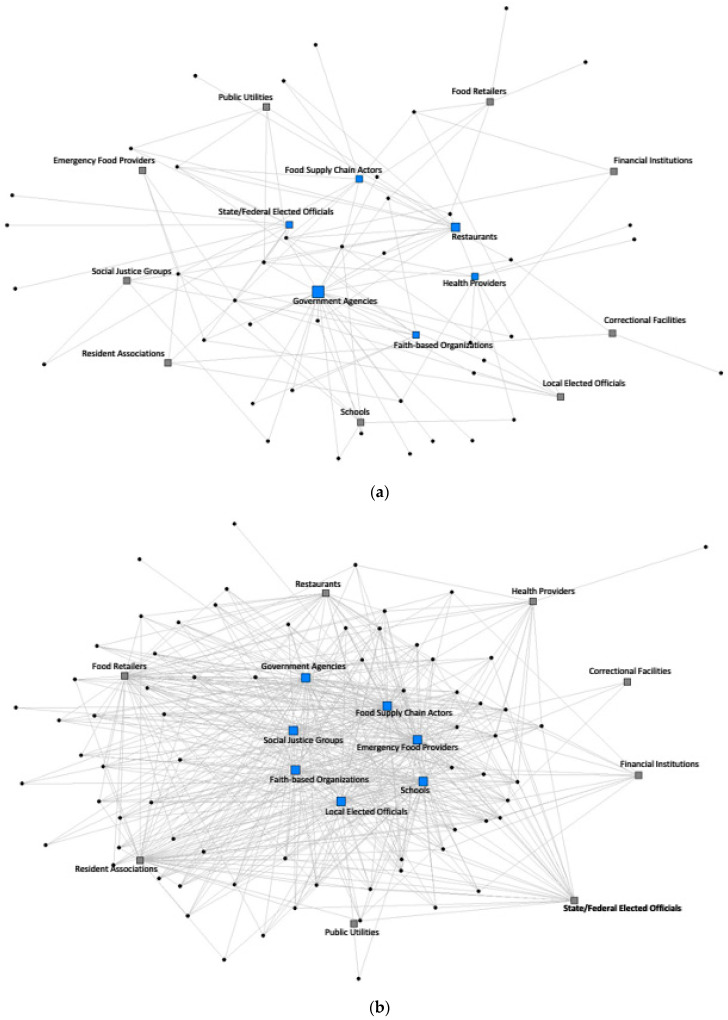
(**a**) Sociogram of core (blue) and peripheral (grey) partners within FPCs using a racial or social equity framework and their new networks. (**b**) Sociogram of core and peripheral partners within FPCs using a racial or social equity framework and their established networks.

Figure 3a,b show sociograms of FPCs’ new and established networks with partners, focusing here on a subset of FPCs that reported using a racial or social equity framework (*n* = 76). In this sub-analysis, we observed that network density in FPCs’ new networks was 10.7% (Figure 3a) versus 50.0% in FPCs’ established networks (Figure 3b). Overlapping core partners in FPCs’ new and established networks included government agencies, faith-based organizations, and food supply chain actors. However, unique core partners only in FPCs’ new networks included healthcare providers, state or federal elected officials, and restaurants. In FPCs’ established networks, social justice groups were core partners, while food retail stores were non-core partners. 

#### 3.2.2. Characteristics of FPCs Network Partnerships, Using a Racial or Social Equity Framework, and Associated Programmatic, Policy, and Advocacy Actions

Overall, only the network characteristics within FPCs’ established networks were significantly associated with a greater likelihood of being responsive to food systems’ needs by leading or supporting programmatic actions, leading or supporting policy changes, and taking on advocacy actions (Table 3). Again, the largest association was observed in relation to network density. For example, every unit increase in network density was associated with a 2.37 greater likelihood of leading or supporting programmatic actions (95% CI 1.58, 3.60; *p* < 0.01), a 5.57 greater likelihood of leading or supporting policy changes (95% CI 2.55, 12.18; *p* < 0.01), and an 8.82 greater likelihood of taking on advocacy actions (95% CI 3.31, 23.49; *p* < 0.01). 

## 4. Discussion

To our knowledge, this is the first study to apply an SNA approach to evaluate how FPCs’ network structures relate to programmatic, policy, and advocacy actions in response to the COVID-19 pandemic. We identified core and peripheral actors in FPCs’ networks and characteristics of FPCs’ network structures that may have enabled them to pivot their work toward responding to food-related needs and demands. 

First, the findings from this study highlight core partners within FPCs’ networks. Not surprisingly, overall (i.e., within FPCs’ established and new networks), we found that government agencies (e.g., health and human/social services, planning, emergency management services) and food supply chain actors (e.g., producers, food chain workers) were prominent in food systems’ responses during the pandemic. Across the country, the pandemic required elected officials and government staff to establish more flexible policies and quickly direct funding and resources to support food-related programming and services [39,40]. Meanwhile, local and regional food supply chain actors made agile pivots to new market channels and buyers by diverting food products to other retail outlets, schools, pantry programs, and more [41]. In these efforts, FPCs often served a matchmaking role by facilitating connections across food system sectors and matching resources with needs, such as supporting connections between producers and new purchasers. 

Faith-based organizations also played a prominent role within FPCs’ networks, particularly among FPCs using a racial or social equity framework. Several researchers have described the importance of the church and faith-based organizations as an ally in efforts to provide health and social services, including food assistance, to marginalized and at-risk populations [42,43,44,45]. Because many faith-based organizations already had established trust with their respective community members as well as the infrastructure in place from overseeing food pantries, they were well positioned during the pandemic to provide networking and logistical support for distributing food assistance, coordinating local projects, and disseminating information related to food drives, donations, and food delivery [46]. The findings from additional studies also document how faith-based organizations mobilize spiritual values to tackle the root causes of food insecurity and diet-related disease [47]. Because faith communities are increasingly focusing on food justice work, they hold promise as key partners in FPCs’ work to advance racial and social equity in food systems. 

Notably, social justice groups were core partners among the sub-set of FPCs using a racial or social equity framework. However, in FPCs’ networks overall, social justice groups were not reportedly core partners. This suggests that FPCs with an explicit focus on racial or social equity were better positioned to establish and sustain relationships with social justice groups when responding to food systems’ needs during the pandemic. A growing number of studies have documented the extent to which the COVID-19 pandemic has magnified existing disparities in food security, disproportionately affecting low-income households and communities of color [2,48,49,50]. Therefore, centering the work of FPCs on confronting racial injustice in the food system is consequential for advancing more equitable and healthy food systems in the long term. To further build FPCs’ capacity to foster new relationships with social justice groups, future research could benefit from a closer examination of how relationships with social justice groups emerge, and how changes in the strength and quality of these relationships over time influence FPCs’ actions and responses to food-related needs. In addition, the important role of food supply chain actors merits more research to better understand how FPCs are engaging with different stakeholders in this group from producers to food chain workers to distributors. Food retail workers, for example, enabled access to food for all Americans during the pandemic, yet many remain some of the most economically vulnerable populations in the food system.

While core actors tend to be involved in more issues than peripheral actors, peripheral actors can play a significant role in more focused food system responses or initiating actions in the network [51]. With regard to peripheral partners in FPCs’ networks overall, we found that public utilities, correctional facilities, banks/financial institutions, and resident associations were less prominent in food systems responses during the pandemic. For example, while FPCs are commonly involved in institutional food procurement policies, such as those involved schools and hospitals, this study suggests that engagement with correctional facilities remains underexplored by FPCs. This gap was made especially salient amid COVID-19. In one of the first national assessments of food experiences in correctional facilities, the report by Impact Justice found a scarcity of fresh produce with 62% of incarcerated people reporting never having access to fresh vegetables and 55% never having access to fresh fruits [52]. Because people of color are disproportionately incarcerated and are more likely to experience food insecurity both before and after incarceration, future efforts could explore stronger partnerships between FPCs and correctional facilities vis-à-vis state-specific policy and funding opportunities impacting food procurement and preparation processes so that food is a source of health, healing, and dignity for incarcerated people [53]. Adoption of the Good Food Purchasing Program in Alameda County, CA, is an example of a landmark resolution to improve food in county correctional facilities and support a more just and sustainable food system [54].

Interestingly, among FPCs using a racial or social equity framework, food retailers were identified as a peripheral partner despite the essential access many food retailers (e.g., grocery stores) provide to healthy and affordable food options. This finding points to opportunities to address challenges around inequitable food environments by strengthening the connections between FPCs and food system sectors more expansively, such as the food retail sector. Since the early months of the pandemic, FPCs have played an essential role in coordinating emergency food assistance, linking food producers to new markets, supporting food chain workers, and more [11]. 

To build on the impact of FPCs going forward, efforts to advance economic justice and development will be essential [55,56,57], and councils are increasingly moving in this direction. For example, the Chicago (IL) Food Policy Action Council advocated for increased funding to support BIPOC-owned and controlled food system organizations and businesses in the provisioning, coordination, preparation, and delivery of COVID-19 relief meals and food box programs. Additionally, the Franklin County (OH) Local Food Council convened listening sessions to draw attention to disproportionately low wages among people of color in the food system. Ultimately, racial and social equity in the food system will be more fully realized when the ownership and control of all food system sectors are reflective of the communities they serve. 

Lastly, our network analysis showed that network density (compared to degree and coreness) had the largest association with any action by FPCs but was especially pronounced for advocacy actions taken by FPCs; we found a similarly positive, though greater, effect size among FPCs using a racial or social equity framework. Prior research has documented positive associations between network density and self-efficacy, well-being, and the flow of goods, services, and support [22,58,59]. Therefore, prioritizing efforts to increase FPC organizational network density (connectedness with partners) may be a promising strategy to advance programmatic, policy, and advocacy actions by facilitating the ability of FPCs and their partners to work together. Additionally, FPCs’ established networks (compared to new networks) were more likely to take action in response to the pandemic. In other words, FPCs with partner relationships that existed prior to the pandemic and existed at the time of the survey had a greater likelihood of responding to food systems’ needs. To further the work and impact of FPCs, future research could evaluate how interconnectedness and longer-lasting relationships with partners develop over time, especially among FPCs with a focus on racial and social equity. Clearer identification of network structures and successful relationship-building strategies can build FPCs’ capacity, leading to more impactful and equitable food system responses [60].

This research offers insights into FPCs’ social networks and related food systems responses in the pandemic. However, the findings from this study should be considered with limitations in mind. The survey data represent a snapshot in time, and survey responses include FPCs believed to be active, in development, or in transition as of June 2020. A follow-up study would benefit from examining changes in FPCs’ social networks over time. The survey was distributed to 372 food policy councils and state conveners of FPCs across the United States between June and September 2020; nearly 46 of these councils had become inactive over the previous two years or did not qualify as an FPC. A further limitation is that the survey was not initially designed to capture social networks and network characteristics. Future work would benefit from more detailed information, such as the duration of network relationships and the frequency and type of interactions between FPCs and their respective partners. Finally, the survey was completed by individuals representing their councils (i.e., key contacts for the FPC). Therefore, the responses may represent individual perspectives and not fully capture FPCs as a whole nor associated organizational perspectives. 

## 5. Conclusions

FPCs have played a critical role in organizing coordinated efforts across multiple sectors to address food systems’ challenges during the COVID-19 pandemic. However, there is limited empirical evidence of the nature of relationships within FPCs and how FPCs’ network structures influence programmatic, policy, and advocacy actions. Using a social network analysis approach as an evaluation tool to identify priorities for improving network function, findings from this study begin to uncover the characteristics of FPCs’ network partnerships that may have facilitated more responsive programmatic, policy, and advocacy actions. In particular, faith-based organizations and social justice groups are likely to be important partners to ensure equitable resource distribution across the food system in an emergency response. Additionally, our results suggest that network density (connectedness) may be more important than other network characteristics, such as degree, when responding to food-related needs and demands during the COVID-19 pandemic. Therefore, in preparation for future emergencies, FPCs may benefit from strengthening connectedness among partners to facilitate an expeditious flow of goods, information, services, and support. Going forward, the work of FPCs could be further improved with more systematic documentation of FPCs’ network partnerships and related change in actions as partnerships evolve to respond to food systems’ needs. In this future research, it would be valuable to also evaluate the social, economic, and political conditions that enable the establishment and connectedness of partnerships within FPCs. 

## Figures and Tables

**Table 1:** Summary characteristics of FPCs (*N* = 198).

	*n* (%)
Regional location	
Midwest	62 (31.5)
West	50 (25.4)
South	48 (24.4)
Northeast	37 (18.8)
Type of organization	
Housed in nonprofit	66 (33.3)
Embedded in government	49 (24.8)
Grassroots coalition	40 (20.2)
Nonprofit	30 (15.2)
Embedded in university	9 (4.6)
Other	4 (2.02)
Geographic focus	
County	74 (37.4)
Both city/municipality and county	38 (19.2)
Region (multi-county or multi-state)	38 (19.2)
City/municipality	24 (12.1)
State/territory	21 (10.6)
Native American or Indigenous lands	3 (1.5)
Annual budget	
None	57 (28.9)
USD 1–10,000	67 (34.0)
USD 10,001–25,000	22 (11.2)
USD 25,001–100,000	30 (15.2)
Over USD 100,000	21 (10.7)

**Table 2:** Characteristics of FPCs’ new and established network partnerships associated with programmatic, policy, and advocacy actions taken in response to the pandemic.

		New Network Partnerships						Established Network Partnerships					
		Programmatic Actions Led/Supported		Policy Changes Led/Supported		Advocacy Actions Taken		Programmatic Actions Led/Supported		Policy Changes Led/Supported		Advocacy Actions Taken	
Model	Network Characteristics	IRR	95% CI	IRR	95% CI	IRR	95% CI	IRR	95% CI	IRR	95% CI	IRR	95% CI
1	Degree	1.02 ^†^	(1.01, 1.04)	1.03	(0.98, 1.09)	1.02	(0.97, 1.08)	1.04 *	(1.03, 1.05)	1.07 *	(1.05, 1.10)	1.08 *	(1.05, 1.11)
2	Coreness	1.05 *	(1.02, 1.09)	1.06	(0.97, 1.17)	1.04	(0.94, 1.15)	1.05 *	(1.03, 1.06)	1.10 *	(1.06, 1.13)	1.09 *	(1.05, 1.13)
3	Density	1.73 ^†^	(1.14, 2.61)	2.06	(0.69, 6.11)	1.63	(0.46, 5.77)	2.37 *	(1.58, 3.57)	5.37 *	(2.86, 10.10)	6.99 *	(3.37, 14.51)

* *p* < 0.01; ^†^ *p* < 0.05.

**Table 3:** Characteristics of FPCs new and established network partnerships, using a racial or social equity framework, and associated programmatic, policy, and advocacy actions taken in response to the pandemic.

		New Network Partnerships						Established Network Partnerships					
		Programmatic Actions Led/Supported		Policy Changes Led/Supported		Advocacy Actions Taken		Programmatic Actions Led/Supported		Policy Changes Led/Supported		Advocacy Actions Taken	
Model	Network Character-istics	IRR	95% CI	IRR	95% CI	IRR	95% CI	IRR	95% CI	IRR	95% CI	IRR	95% CI
1	Degree	1.01	(0.98, 1.03)	1.02	(0.96, 1.08)	1.02	(0.95, 1.1)	1.03 *	(1.01, 1.05)	1.07 *	(1.04, 1.10)	1.08 *	(1.05, 1.12)
2	Coreness	1.01	(0.98, 1.05)	1.01	(0.93, 1.09)	1.03	(0.93, 1.13)	1.02 ^†^	(1.00, 1.03)	1.05 *	(1.02, 1.07)	1.06 *	(1.03, 1.10)
3	Density	1.20	(0.69, 2.08)	1.35	(0.36, 5.05)	1.57	(0.32, 7.86)	2.37 *	(1.58, 3.60)	5.57 *	(2.55, 12.18)	8.82 *	(3.31, 23.49)

* *p* < 0.01; ^†^ *p* < 0.05.

## Data Availability

The data presented in this study are available on request from the corresponding author. The data are not publicly available due to privacy restrictions.

## Supplementary Materials

The following supporting information can be downloaded at: https://www.mdpi.com/article/10.3390/nu16070915/s1, survey portion reflecting select questions that were used for this study.
