# Supplementary material for: Characterizing Food Policy Councils’ Network Partnerships and COVID-19 Responses
Source: Nutrients. 2024 Mar 22;16(7):915. doi: 10.3390/nu16070915 (PMC11013245; doi:10.3390/nu16070915)
Supplement: Supplementary file 1 [file nutrients-16-00915-s001.zip › nutrients-2897082-supplementary.pdf]

Supplementary Materials

The following portion of the survey reflects select questions that were used for this study.

Does your FPC work with any of the following organizations, agencies or people on food systems concerns related to COVID-19?

**Organization**	**FPC previously worked with group and is currently working with group**	**New relationship for FPC**	**No relationship with group**
1) Government agency - Emergency management services			
2) Government agency - Planning department			
3) Government agency - Parks and recreation department			
4) Government agency - School district			
5) Government agency - Transportation department			
6) Government agency - Health and human or social services			
7) Government agency - Police department			
8) Government agency – Agriculture			
9) Government agency – Environment or Sustainability			
10) Local elected official's office (e.g., mayor, city council, county commission)			
11) State elected official's office (e.g., governor, state representative or senator)			
12) Federal elected official's office (e.g. staff member or aide for Senator or Representative)			
13) Public utilities			
14) Emergency food providers (e.g., food bank, food pantry, hot meal provider)			

15) Faith-based organizations			
16) Restaurants			
17) Food retail stores (e.g. grocery or convenience stores, cooperative markets)			
18) Producers or direct markets for producers (e.g., farmers, ranchers, watermen, farmers markets, CSAs, farm bureau, farmer cooperatives)			
19) Food chain workers (e.g. labor unions or collectives)			
20) Food processors, distributors or suppliers (e.g. truck drivers, meat processing facilities)			
21) Correctional facilities			
22) Healthcare providers (e.g., hospitals, community clinics)			
23) Frontline healthcare workers and first responders (i.e. EMTs)			
24) Social justice or civil rights advocacy groups			
25) Assisted living facilities and nursing homes			
26) Banks, financial institutions or loan services			
27) Elementary and secondary schools			
28) Colleges, universities or community colleges			
29) Residents or neighborhood associations			

Does your FPC use a racial or social equity framework or criteria when making decisions about its policy priorities or advocacy actions?

a. Yes
b. No
c. Developing criteria or framework

What actions has your FPC taken in response to COVID-19?

**Action**	**Our FPC led this effort**	**Our FPC supported another organization's efforts**	**Our FPC has not taken action in this area**
1) Created a publicly accessible webpage with food systems information (e.g. resources, services) related to COVID-19			
2) Created a map of food systems resources available in response to COVID-19			
3) Created a dashboard to track food systems related trends in response to COVID-19			
4) Used social media (e.g., Twitter and Facebook) to share real-time information about the status of food systems resources during COVID-19			
5) Held community meeting(s) related to COVID-19			
6) Collected data related to food and farm systems needs due to COVID-19			
7) Held regular meetings between food systems sector representatives about responses to COVID-19			
8) Facilitated connections across food systems sectors to match resources with needs to respond to COVID-19			
9) Educated the public about food systems challenges due to COVID-19			
10) Advocated for local, state, tribal or federal policies related to community food and farm systems concerns due to COVID-19			

11) Met with government staff and/or elected officials to discuss food systems issues related to COVID-19			
12) Worked with government staff and/or elected officials to provide funding for needed food systems services in response to COVID-19			
13) Worked with community partners to raise funds for needed food systems services in response to COVID-19			

Please describe other actions your FPC has taken in response to COVID-19 that were not listed in the chart above:

Which areas has your FPC helped to develop or advocated for policies in response to COVID-19, either directly or by supporting efforts led by another organization?

	**Our FPC led or supported policy changes in this issue area**	**Did not work on policy changes in this issue area**
1) Emergency food providers (e.g., food banks, pantries)		
2) Food producers (e.g., farmers, ranchers, watermen)		
3) Food chain workers and other essential workers (e.g., farmworkers, processing workers, food service workers)		
4) Direct markets for local producers (e.g., farmers markets, farm stands or CSAs)		
5) Federal food and nutrition assistance programs		
6) Federal child nutrition programs (e.g., school meals, summer meals, WIC, SNAP)		

7) Food processing and distribution		
8) Food procurement		
9) Food safety		
10) Restaurants and food service establishments		
11) Food retail stores		
12) Institutions (e.g., schools, hospitals, jails)		
13) Urban agriculture or community gardens		
14) Access to credit or Small Business Administration loan programs		
15) Funding for critical food systems needs		

Please describe other areas that your FPC has helped to develop or advocated for policies in response to COVID that were not listed in the chart above:

What advocacy actions has your FPC taken in response to COVID-19?

1) Provided policy recommendations to policy makers
2) Supported or directed a campaign to advocate for a specific policy change
3) Submitted written testimony
4) Submitted comments on regulatory changes
5) Provided oral testimony
6) Met with policy makers
7) Made calls to policy makers
8) Reviewed and commented on draft legislation
9) Supported a partner organization's policy agenda by signing onto a letter or providing testimony
10) Surveyed policymakers about food system issue(s)
11) Educated public about policy issues or candidates
12) Hosted candidate forum
13) Provide analysis or research on specific legislation
14) Other (please explain): ________
15) None (only select if none of the above options selected)
